# Prevalence and predictors of recreational drug use among medical and nursing students in Cameroon: a cross sectional analysis

**DOI:** 10.1186/s13104-018-3631-z

**Published:** 2018-07-28

**Authors:** Clarence M. Mbanga, Derrick T. Efie, Desmond Aroke, Tsi Njim

**Affiliations:** 1 Mankon Sub Divisional Hospital, Bamenda, Cameroon; 2 Tokombéré District Hospital, Tokombéré, Cameroon; 3 Health and Human Development Research Network, Douala, Cameroon; 4 Fontem District Hospital, Fontem, Cameroon

**Keywords:** Recreational drug use, Medical students, Nursing students, Cameroon

## Abstract

**Objective:**

Medical and nursing students in Cameroon are likely to have mental health problems given the stressful nature of their studies. Paucity of mental health institutions in the country implies they hardly get access to professional help when needed and are obliged to develop coping strategies such as recreational drug use. This study aims to determine the prevalence and predictors of recreational drug use among a group of Cameroonian medical and nursing students.

**Results:**

Cross-sectional analysis of 852 medical and nursing students (mean age 21.78 ± 3.14, 31.49% males) recruited by convenience sampling from three state-owned medical schools; and from two state-owned and two private nursing schools in Cameroon over a four-month period (January–April 2018). Information was collected via a printed self-administered and structured questionnaire from consenting students. Multivariable logistic regression analysis was used to identify independent predictors of recreational drug use. The overall prevalence of recreational drug use was 1.64% with tramadol and marijuana noted as the drugs used by these students. Independent predictors of recreational drug use were: presence of a chronic illness (OR 5.26; 95% CI 1.32, 20.97; p = 0.019), alcohol consumption (OR 5.08; 95% CI 1.54, 16.73; p = 0.008) and Total Oldenburg Burnout Inventory score (OR 1.11; 95% CI 1.02, 1.21; p = 0.021). The use of recreational drugs by medical and nursing students in Cameroon remains worrisome despite its very low prevalence, as it may negatively impact their performance and health.

**Electronic supplementary material:**

The online version of this article (10.1186/s13104-018-3631-z) contains supplementary material, which is available to authorized users.

## Introduction

Recreational drugs are legal and illegal drugs used without medical supervision. The four categories of recreational drugs include analgesics, depressants, stimulants, and hallucinogens [1]. The increasing use of recreational drugs among students has become a rising and disturbing phenomenon worldwide [2, 3].

This uprising problem is relevant in Cameroon. A rapid assessment on drug abuse in Cameroon carried out in 1996, reported that males and females of all age groups were involved in the consumption of recreational drugs [3]. According to the report, narcotics consumed in Cameroon ranged from Cannabis (Marijuana) which was the most consumed, to traditional drugs, a host of other pharmaceutical drugs, as well as cocaine and heroin [3].

Consumption of marijuana and other stupefying drugs has been reported amongst medical and nursing students elsewhere [4]. The prevalence of recreational drug use among students in the health sector generally ranges from 2% to as high as 64% [5–8]. The reasons for which students consume recreational drugs include pleasure and relaxation, peer influence, cigarette smoking, and heavy drinking [9, 10].

Medical and nursing students have been shown to have high rates of mental health disorders such as burnout, stress, anxiety and depression [11–14]. Several reports suggest that stressed and depressed students, tend to use alcohol and other recreational drugs more often as a coping strategy [15, 16].

There is dearth of data regarding recreational drug use in Cameroon as a whole, and among students of the health care sector in particular. In order to bridge this gap, we set out to determine the prevalence and predictors of recreational drug use in a group of medical and nursing students from state-owned and private medical and nursing institutions in the country.

## Main text

### Study population

Study participants included 413 medical students from three randomly selected medical schools (University of Bamenda, University of Douala and University of Buea and 447 nursing students from the two-state owned (University of Bamenda and University of Buea) and two private nursing institutions (Saint Veronica higher institute of biomedical and nursing sciences Buea and Saint Louis higher institute of health Bamenda). Participants were recruited for the purpose of a greater project aimed at assessing burnout and depression among medical and nursing students in Cameroon and some of the methods have been published in a previous project [17]. A total of 860 medical and nursing students were recruited by a convenience approach.

### Study design and protocol

This was a cross-sectional analysis carried out over a period of 4 months (January 2018–April 2018) using medical and nursing students recruited from the above-mentioned institutions. Students were approached and the nature and purpose of the study explained to them individually. Students who agreed to participate in the study signed a written consent form, and were then handed a printed, structured questionnaire (in English or French depending on their language preferences) to fill and return at their earliest convenience.

### Study instrument

The study instrument was a printed structured questionnaire made up of three sections. The first section (Additional files 1, 2) sought the following socio-demographic characteristics: age; sex; marital status; presence and number of (children) dependents; level of studies; number of hours spent studying; presence of problems in personal relationships (defined as a close connection between two people formed by emotional and sexual interactions); monthly income and the perception of sustainability on their monthly income; average academic results attained; alcohol use; recreational drug use and type; smoking and history of any chronic disease.

The second section assessed burnout syndrome using the Oldenburg Burnout Inventory (OLBI), consisting of 16 positively and negatively framed questions which assesses two key components of burnout: exhaustion (intense physical, affective and cognitive strain) and disengagement (characterized as cynicism referring to distancing from one’s work in general) [18]; with eight questions for each parameter. We used the version of the OLBI questionnaire adapted for use in an academic milieu by Reis et al. [19].

The third section assessed depression using the simple yet accurate Patient Health Questionnaire-9 (PHQ-9) [20]. Validated versions of both the OLBI and PHQ-9 in French [21, 22], were pre-tested before use amongst participants with a French orientation.

### Data management and statistical analysis

Data from returned questionnaires was entered into EPI Info version 7 (CDC, Atlanta) and later exported to the Stata software package version 12 (Statacorp, College Station, TX, USA) for statistical analysis.

Both subscales of the OLBI (emotional exhaustion and cynicism) were added to obtain a total score for burnout syndrome, after negatively phrased questions were reversed (1 = 4, 2 = 3, 3 = 2, 4 = 1) such that higher scores indicated higher exhaustion and disengagement in the students.

Results were presented as counts (percentages), mean and standard deviation (SD).

Univariable analysis was performed to determine predictors of recreational drug use. Variables significant on univariable analysis were inputted into a multivariable logistic regression model to determine independent predictors of recreational drug use. Significance was set at 5%.

## Results

### General characteristics

Of the 860 students who filled the questionnaire, 852 provided an answer to the question on recreational drug use (99.07% response rate) and were used for the final analysis. Participants had a mean age of 21.78 ± 3.14 years (range 16–50 years), and were predominantly female (68.51%). Most of the students (70.45%) were from the state-owned universities and a majority were single (93.25%) (Table 1). The mean total OLBI score was 37.58 ± 5.37 while the mean total PHQ-9 score was 6.92 ± 4.32 (Table 2).

**Table 1:** Categorical variables showing the sociodemographic characteristics of 852 medical and nursing students from January–April 2018

Variable	Total	
	N	%
University (n = 846)		
University of Bamenda	256	30.25
University of Buea	259	30.62
University of Douala	81	9.58
Private sector	250	29.55
Gender (n = 851)		
Male	268	31.49
Female	583	68.51
Personal relationship (n = 844)^a^		
Yes	383	45.38
No	461	54.62
Marital status (n = 845)		
Single	788	93.25
Married	57	6.75
Difficulties in personal relationship (n = 788)		
Yes	153	19.42
No	635	80.58
Exams re-sited (n = 828)		
Yes	453	54.71
No	375	45.29
Courses repeated (n = 833)		
Yes	329	39.50
No	504	60.50
Satisfaction with results (n = 772)		
Yes	223	28.89
No	549	71.11
Regret choice of studies (n = 842)		
Yes	84	9.98
No	758	90.02
Occurrence of life changing crises in last 6 months (n = 830)^b^		
Yes	378	45.54
No	452	54.46
Presence of chronic illness (n = 851)^c^		
Yes	38	4.47
No	813	95.53
Alcohol consumption (n = 851)		
Yes	248	29.14
No	603	70.86
Recreational drug use (n = 852)^d^		
Yes	14	1.64
No	838	98.36
Sufficient monthly income (n = 794)		
Yes	253	31.86
No	541	68.14

^a^Personal relationship was defined as close connections between two people formed by emotional and sexual interactions

^b^Life changing crises defined as loss of a loved one, physical or sexual trauma and conditions of emotional or social instability

^c^Chronic illnesses included: asthma, chronic pelvic pain, diabetes mellitus, gastroesophageal reflux disease, chronic peptic ulcer disease, migraines, cerebral lesions and paralysis

^d^Recreational drugs included: marijuana and tramadol

**Table 2:** Continuous variables showing the sociodemographic characteristics of 852 medical and nursing students from January–April 2018

Variable	Number of observations	Total sample			
		Mean	SD	Min	Max
Age	767	21.78	3.14	16	50
Number of hours studying	806	4.31	2.75	0	19
Monthly income in USD	551	40.35	33.13	0	281.52
GPA	614	2.82	0.54	0	4
Total OLBI score	852	37.58	5.37	21	63
Total PHQ-9 score	852	6.92	4.32	0	25

*USD* United States dollars, *GPA* cumulative grade point average, *OLBI* Oldenburg Burnout Inventory, *PHQ*-*9* Patient Health Questionnaire-9

### Prevalence of recreational drug use

Fourteen students admitted to using recreational drugs (Tramadol and marijuana) giving a prevalence of 1.64% (95% CI 0.98–2.74%).

### Predictors of recreational drug use

On univariable analysis, being in a personal relationship (OR 3.06; 95% CI 0.87, 13.47; p = 0.048); presence of a chronic illness (OR 6.25; 95% CI 1.07, 25.02; p = 0.002) and alcohol consumption (OR 6.29; 95% CI 1.79, 27.68; p < 0.001) were associated with recreational drug use (Table 3). The Total OLBI score (p = 0.007) was also associated with recreational drug use (Additional file 3).

**Table 3:** Univariable analysis for potential categorical predictors of recreational drug use among 852 medical and nursing students in Cameroon from January–April 2018

Variable	Category	Total	n	%	OR	95% CI	p
Gender (n = 851)	Female	583	11	1.89	1.70	0.44, 9.55	0.414
	Male	268	3	1.12			
Marital status (n = 845)	Married	57	1	1.75	1.07	0.03, 7.34	0.952
	Single	788	13	1.65			
Personal relationship (n = 844)^a^	Yes	383	10	2.61	3.06	0.87, 13.47	0.048
	No	461	4	0.87			
Difficulties in personal relationship (n = 788)	Yes	153	5	3.27	2.65	0.67, 9.32	0.080
	No	635	8	1.26			
Monthly income sufficient (n = 794)	Yes	253	5	1.98	1.34	0.34, 4.71	0.607
	No	541	8	1.48			
Course repeated (n = 833)	Yes	329	9	2.74	2.81	0.84, 10.75	0.056
	No	504	5	0.99			
Exam re-sited (n = 828)	Yes	453	11	2.43	3.09	0.81, 17.33	0.070
	No	375	3	0.80			
Satisfaction with results (n = 772)	Yes	223	5	2.24	1.55	0.40	0.442
	No	549	8	1.46			
Regret choice of studies (n = 842)	Yes	84	2	2.38	1.51	0.16, 6.99	0.587
	No	758	12	1.58			
Life changing crises (n = 830)^b^	Yes	378	6	1.59	1.03	0.28, 3.60	0.964
	No	452	7	1.55			
Presence of chronic illness (n = 851)^c^	Yes	38	3	7.89	6.25	1.07, 25.02	0.002
	No	813	11	1.35			
Alcohol consumption (n = 851)	Yes	248	10	4.03	6.29	1.79, 27.68	< 0.001
	No	603	4	0.66			

^a^Personal relationship was defined as close connections between two people formed by emotional and sexual interactions

^b^Life changing crises defined as loss of a loved one, physical or sexual trauma and conditions of emotional or social instability

^c^Chronic illnesses included: asthma, chronic pelvic pain, diabetes mellitus, gastroesophageal reflux disease, chronic peptic ulcer disease, migraines, cerebral lesions and paralysis

^d^Recreational drugs included: marijuana and tramadol

On multivariable analysis, the following variables were shown to be independent predictors of recreational drug use: presence of a chronic illness (OR 5.26; 95% CI 1.32, 20.97; p = 0.019), alcohol consumption (OR 5.08; 95% CI 1.54, 16.73; p = 0.008) and Total OLBI score (OR 1.11; 95% CI 1.02, 1.21; p = 0.021) (Additional file 4).

## Discussion

Our results indicate a prevalence of recreational drug use of 1.64% in a combination of nursing and medical students, and identified the presence of a chronic illness, alcohol consumption and burnout syndrome assessed using the total OLBI score as independent predictors of recreational drug use among this population (Additional file 5).

Data on recreational drug use in Cameroon is sparse. However, there have been reports on the increasing use and abuse of the analgesic and opioid drug, tramadol, by youths in the North west region of the country, with the primary aim of trying to achieve mood and performance enhancement [23]. Marijuana alongside a large range of pharmaceutical drugs remain the most abused substances by youths in Cameroon according to certain reports [3]. This was reflected in our findings, with tramadol and marijuana being the only recreational drugs listed by participants who confirmed taking recreational drugs.

We obtained a prevalence of recreational drug use among nursing/medical students of 1.64%. The prevalence recorded in our study was considerably lower than that reported elsewhere [5, 6, 8, 24]. This could be attributable to under reporting as recreational drug use is considered a taboo in this conservative society. Also, the Kübler-Ross model (known as the five stages of grief) could be applicable in drug addiction treatment [25]. As such, the first phase recreational drug users or abusers go through in an attempt to come to terms with their addiction problem is denial. It is probable that only a few students using recreational drugs were past the denial phase and felt confident enough to report on the questionnaire.

Medical and nursing studies in Cameroon are quite stressful and time consuming, with students having to attend several hours of lectures daily. In addition, students are faced with the psychological and emotional challenge that comes with seeing and caring for critically ill and dying patients during internships. Dealing with the challenges that come along with managing a chronic disease alongside the stress from studies could be quite an uphill task. It is therefore not surprising that students faced with such a double dose of burden seek for a coping strategy, one of which is the use of recreational drugs [15, 16]. This is worsened by the fact that mental health institutions and professionals are scarce in our setting, making it difficult for those students who recognise their need for professional help to get one. Recreational drug use could also worsen the course of the ongoing chronic illness, and negatively influence adherence to treatment [25, 26]. The end result is a student who finds themselves in a downward spiral in which stress resulting from the challenges of studies and managing a chronic illness drives them into drug use, drug use worsens their disease and or compliance to their treatment and ultimately their health, poor health translates into poor performance which may lead to more stress and further drug use.

Alcohol intake has been previously associated to recreational drug use and abuse, with certain studies indicating that individuals reporting frequent alcohol intake are about five times more likely to use recreational drugs [9, 10]. Alcohol is regarded as a gateway drug. According to the gateway theory, the use of a psychoactive or addictive drug increases the likelihood of using other drugs [27]. Translating this in the context of our study, the intake of alcohol (a well-known addictive substance) increases the chances of use and/or abuse of other addictive drugs such as marijuana or tramadol. Some of the mechanisms put forth to explain this theory include; the possible biological alterations in the brain due to the earlier drug which increases the chances of trying out another drug, and the similarity in the personalities of users of different drugs which makes more likely for the user of one drug to readily use another drug [28]. The association that could possibly exist between alcohol intake and recreational drug use is even more highlighted in a special population like medical/nursing students, where mental health problems exist at high levels [11–14]. Students faced with mental health problems often use alcohol intake as a coping strategy [15, 16]. Alcohol intake as shown in our results, predisposes them to recreational drug use. This highlights the need for early detection of students at risk and the proper management of those already using, reiterating the need for equipped mental health facilities and properly trained mental health personnel in our health training institutions.

Burnout syndrome is relatively common among students in the health care sector [11, 14]. Entrance exams into the state-owned medical and nursing schools in Cameroon are usually very competitive with limited spots available for grasp. Those who do not make it are left no other option but fight for a spot in the remaining private institutions whose tuition fees are considerably higher. Those who make it could easily get overwhelmed from the challenges involved in adapting to life in the university, adapting to the relatively new concept of the basic sciences, as well as the demanding, stressful and time-consuming nature of medical or nursing studies as a whole as stated above. All these, alongside the realization of the tremendous workload that awaits them upon completion, could readily culminate to burnout.

There is an overall paucity in the number of mental health facilities and properly trained mental health personnel in Cameroon. Consequently, special populations like medical/nursing students at risk of mental health problems such as burnout do not have access to adequate professional help and care when need arises. This further exposes them to various coping strategies, some of which (recreational drug use) may be inappropriate and detrimental to their health and performance. This highlights the need for more attention on the mental health status of students in health training facilities, in order to provide help for recreational drug users and to screen for and manage mental health problems detected early-on in this at risk population.

### Conclusion

Though the prevalence of recreational drug use among medical and nursing students in Cameroon is relatively low, their use remains worrisome as it could be detrimental to both their health and performance. Predictors herein require further investigation in order to ease early identification and management of students at higher risk of recreational drug use, and to provide data needed for the reform of certain mental health policies in Cameroon.

## Limitations

The cross-sectional nature of this study made it difficult to determine temporal associations between predictors such as alcohol intake, burnout syndrome and recreational drug use. We therefore cannot ascertain the direction of these relationships. We therefore recommend prospective studies to better and properly assess this relationship. Also, the frequency of drug use by participants was not assessed in this study. More comprehensive studies are required to further confirm the results of this study and assess the scale of this problem in this population. Given the sensitive nature of the topic in a highly conservative society and the fact that we had no control over what students reported on their questionnaires, it is possible that students may have underreported their use of recreational drugs to conform with societal expectations.

## Additional files

**Additional file 1.** Section A of questionnaire for medical students. First part of data collection tool used to obtain sociodemographic characteristics of medical students.

**Additional file 2.** Section A of questionnaire for nursing students. First part of data collection tool used to obtain sociodemographic characteristics of nursing students.

**Additional file 3.** Univariable analysis for continuous variables. Univariable analysis for potential continuous predictors of recreational drug use among 852 medical and nursing students in Cameroon from January–April 2018.

**Additional file 4.** Multivariable analysis for independent predictors. Multivariable logistic regression analysis for independent predictors of recreational drug use among 852 medical and nursing students in Cameroon from January–April 2018.

**Additional file 5.** Post hoc analyses. Post hoc analyses showing the interaction between the various predictors (chronic illness, alcohol consumption and burnout syndrome) and the outcome—recreational drug use among 852 medical and nursing students in Cameroon from January–April 2018.

## Abbreviations

OLBI: Oldenburg Burnout Inventory
PHQ-9: Patient Health Questionnaire-9

## References

[1] Hawkes N (2016). Sixty seconds on…psilocybin. BMJ.

[2] Emmons KM, Wechsler H, Dowdall G, Abraham M (1998). Predictors of smoking among US college students. Am J Public Health.

[3] Wansi E, Sam-Abbenyi A, Befidi-Mengue R, Enyme FN, Ntone FN (1996). Rapid assessment of drug abuse in Cameroon. Bull Narc.

[4] Kowalczuk K, Krajewska-Kułak E (2017). Exposure to psychoactive compounds amongst students of Medical University. Cent Eur J Public Health.

[5] Kenna GA, Wood MD (2004). Substance use by pharmacy and nursing practitioners and students in a northeastern state. Am J Health Syst Pharm.

[6] Ahmadi J, Maharlooy N, Alishahi M (2004). Substance abuse: prevalence in a sample of nursing students. J Clin Nurs.

[7] Ahmadi J, Fallahzadeh H, Salimi A, Rahimian M, Salehi V, Khaghani M (2006). Analysis of opium use by students of medical sciences. J Clin Nurs.

[8] Shyangwa PM, Joshi D, Lal R (2007). Alcohols and other substance use/abuse among junior doctors and medical students in a teaching institute. J Nepal Med Assoc..

[9] Barrett SP, Darredeau C, Pihl RO (2006). Patterns of simultaneous polysubstance use in drug using university students. Hum Psychopharmacol Clin Exp..

[10] Panthee B, Panthee S, Gyawali S, Kawakami N (2017). Prevalence and correlates of substance use among health care students in Nepal: a cross sectional study. BMC Public Health..

[11] Da Silva RM, Goulart CT, Lopes LFD, Serrano PM, Costa ALS, de Azevedo Guido L (2014). Hardy personality and burnout syndrome among nursing students in three Brazilian universities-an analytic study. BMC Nurs..

[12] Wahed WAY, Hassan SK (2017). Prevalence and associated factors of stress, anxiety and depression among medical Fayoum Universsity students. Alex J Med..

[13] Tung YJ, Lo KKH, Ho RCM, Tam WSW (2018). Prevalence of depression among nursing students: a systematic review and meta-analysis. Nurse Educ Today.

[14] De Cavalcante Almeida G, de Souza HR, de Almeida PC, de Cavalcante Almeida B, Almeida GH (2016). The prevalence of burnout syndrome in medical students. Arch Clin Psychiatry..

[15] Nwobi EA, Ekwueme OC, Ezeoke EA (2009). Mental depression and coping strategies among medical students of University of Nigeria, Enugu campus. Int J Med Health Dev..

[16] Kumar R (2011). Stress and coping strategies amongst nursing students. Nurs Midwifery Res J..

[17] Njim T, Mbanga C, Mouemba M, Makebe Toukam L, Kika B (2018). Determinants of burnout syndrome among nursing students in Cameroon: cross-sectional study. BMC Res Notes..

[18] Demerouti E, Bakker AB. The Oldenburg Burnout Inventory: a good alternative to measure burnout and engagement. In: Handbook of Stress and Burnout in Health Care; 2008.

[19] Reis D, Xanthopouloub D, Tsaousisc I (2015). Measuring job and academic burnout with the Oldenburg BurnoutInventory (OLBI): factorial invariance across samples and countries. Burnout Res..

[20] Kroenke K, Spitzer RL, Williams JB (2001). The pHQ-9: validity of a brief depression severity measure. J Gen Intern Med.

[21] Arthurs E, Steele RJ, Hudson M, Baron M, Thombs BD, Canadian Scleroderma Research G (2012). Are scores on English and French versions of the PHQ-9 comparable? An assessment of differential item functioning. PLoS ONE.

[22] Arnaud A, Issabelle H. Structural confirmation of the French version of the Oldenburg Burnout Inventory (OLBI). http://hdl.handle.net/2268/149404. Accessed 14 May 2018.

[23] Cameroon-info.Net. Cameroon. Alarming Consumption of Tramadol in the North West Region. http://www.cameroon-info.net/article/cameroon-alarming-consumption-of-tramadol-in-the-north-west-region-297690.html. Accessed 12 May 2018.

[24] Arora A, Kannan S, Gowri S, Choudhary S, Sudrasanan S, Khosla PP (2016). Substance abuse amongst the medical graduate students in a developing country. Indian J Med Res.

[25] Chambers RA, Wallingford SC (2017). On mourning and recovery: integrating the stages of grief and change towards a neuroscience-based model of attachment adaptation in addiction treatment. Psychodyn Psychiatry..

[26] Sansone RA, Sansone LA (2008). Alcohol/substance misuse and treatment nonadherence: fatal attraction. Psychiatry Edgmont..

[27] Steele MR, Belostotsky V, Lau KK (2012). The dangers of substance abuse in adolescents with chronic kidney disease: a review of the literature. Cannt J..

[28] Vanyukov MM, Tarter RE, Kirillova GP, Kirisci L, Reynolds MD, Kreek MJ (2012). Common liability to addiction and “gateway hypothesis”: theoretical, empirical and evolutionary perspective. Drug Alcohol Depend.

