# Supplementary material for: Prevalence and predictors of recreational drug use among medical and nursing students in Cameroon: a cross sectional analysis
Source: BMC Res Notes. 2018 Jul 28;11:515. doi: 10.1186/s13104-018-3631-z (PMC6064166; doi:10.1186/s13104-018-3631-z)
Supplement: Supplementary file 2 — Additional file 2. Section A of questionnaire for nursing students. First part of data collection tool used to obtain sociodemographic characteristics of nursing students. [file 13104_2018_3631_MOESM2_ESM.docx]

**Questionnaire number (For office use only):**

**SOCIODEMOGRAPHIC CHARACTERISTICS**

|  | **VARIABLE** | **CATEGORY** |  |
| --- | --- | --- | --- |
| 1 | Nursing school | 0 = University of Bamenda, 1 = University of Buea, 2 = Other |  |
| 2 | Age |  |  |
| 3 | Gender | 0 = Male, 1 = Female |  |
| 4 | Marital status | 0 = Single, 1 = Married |  |
| 5 | Are you in a relationship? | 0 = no, 1 = yes |  |
| 6 | Do you have problems in your relationship? | 0 = no, 1 = yes |  |
| 7 | Number of children (if any) |  |  |
| 8 | Level of studies | 0 = Year One, 1 = Year Two, 2 = Year Three, 3 = Year Four |  |
| 9 | Number of hours spent studying a day |  |  |
| 10 | Monthly income |  |  |
| 11 | Do you think the amount of money you receive monthly is enough for your needs? | 0 = no, 1 = yes |  |
| 12 | Have you ever repeated a course(s)? | 0 = no, 1 = yes |  |
| 13 | Have you ever had a re-sit exam? | 0 = no, 1 = yes |  |
| 14 | Please give an estimate of your average GPA |  |  |
| 15 | Are you satisfied with your results? | 0 = no, 1 = yes |  |
| 16 | Do you regret choosing to study Nursing? | 0 = no, 1 = yes |  |
| 17 | Have you had any major life changing crises? | 0 = no, 1 = yes |  |
| 18 | Do you have any chronic medical illness? | 0 = no, 1 = yes |  |
| 19 | If yes, which? |  |  |
| 20 | Do you consume alcohol? | 0 = no, 1 = yes |  |
| 21 | How much alcohol do you consume a week? | State type (for example: beer, wine) and number of litres |  |
| 22 | Do you take any recreational drugs? | 0 = no, 1 = yes |  |
| 23 | If yes, state the type of recreational drug |  |  |
