# Supplementary material for: Prevalence and predictors of recreational drug use among medical and nursing students in Cameroon: a cross sectional analysis
Source: BMC Res Notes. 2018 Jul 28;11:515. doi: 10.1186/s13104-018-3631-z (PMC6064166; doi:10.1186/s13104-018-3631-z)
Supplement: Supplementary file 3 — Additional file 3. Univariable analysis for continuous variables. Univariable analysis for potential continuous predictors of recreational drug use among 852 medical and nursing students in Cameroon from January–April 2018. [file 13104_2018_3631_MOESM3_ESM.docx]

| Variable |  | Recreational drug use | | | No recreational drug use | | | p value |
| --- | --- | --- | --- | --- | --- | --- | --- | --- |
|  | N | n | Mean | SD | N | Mean | SD |  |
| Age | 767 | 13 | 21.39 | 3.80 | 754 | 21.79 | 3.13 | 0.649 |
| Monthly income in USD | 551 | 12 | 56.31 | 28.30 | 539 | 40.00 | 33.17 | 0.092 |
| Number of children | 708 | 12 | 0.25 | 0.62 | 696 | 0.16 | 0.58 | 0.576 |
| Cumulative GPA | 614 | 9 | 2.52 | 0.35 | 605 | 2.83 | 0.54 | 0.088 |
| Total OBLI score | 852 | 14 | 41.43 | 9.57 | 838 | 37.52 | 5.26 | 0.007 |
| Number of hours spent studying | 806 | 13 | 3.23 | 2.59 | 793 | 4.33 | 2.75 | 0.153 |
| Total PHQ-9 score | 852 | 14 | 8.21 | 4.35 | 838 | 6.90 | 4.32 | 0.259 |

USD: United states dollars; GPA: cumulative grade point average; OLBI: Oldenburg burnout inventory; PHQ-9: Patient health questionnaire – 9.
