# Supplementary figures and images for: Prevalence and predictors of recreational drug use among medical and nursing students in Cameroon: a cross sectional analysis
Source: BMC Res Notes. 2018 Jul 28;11:515. doi: 10.1186/s13104-018-3631-z (PMC6064166; doi:10.1186/s13104-018-3631-z)

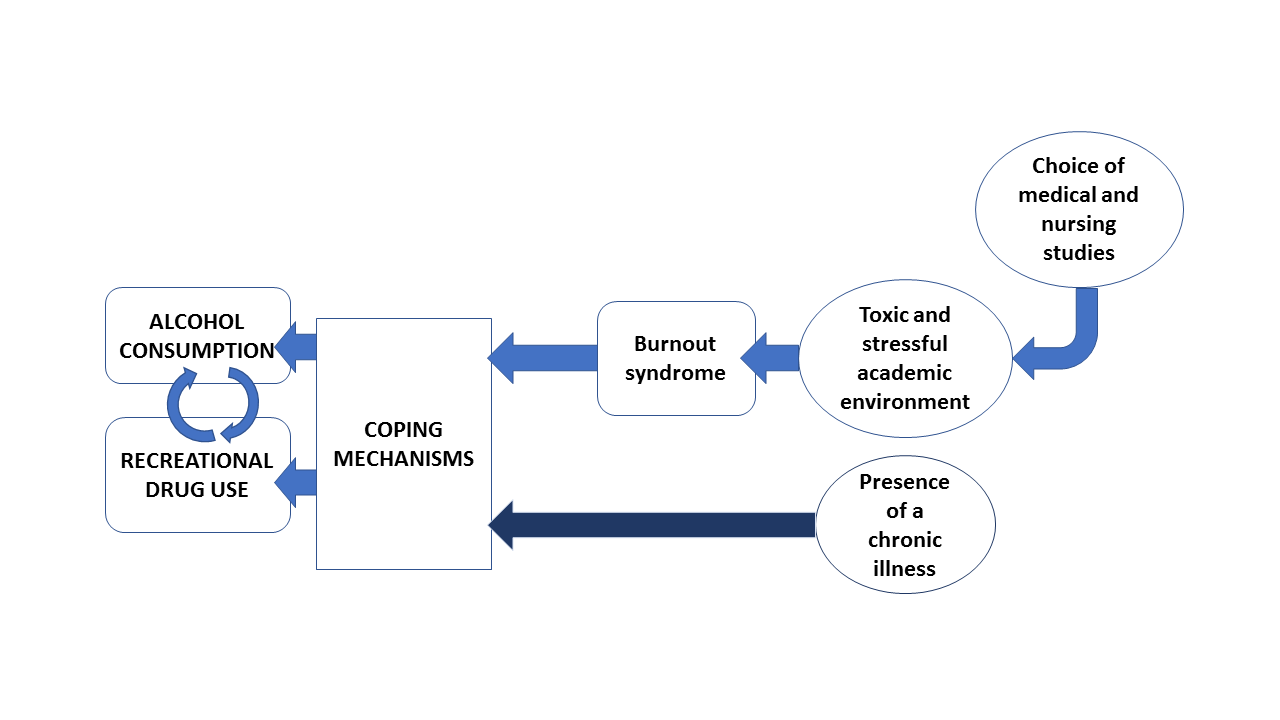

Supplement: Supplementary file 4 — Additional file 4. Multivariable analysis for independent predictors. Multivariable logistic regression analysis for independent predictors of recreational drug use among 852 medical and nursing students in Cameroon from January–April 2018. [file 13104_2018_3631_MOESM4_ESM.png]
