# Supplementary material for: Prevalence and predictors of recreational drug use among medical and nursing students in Cameroon: a cross sectional analysis
Source: BMC Res Notes. 2018 Jul 28;11:515. doi: 10.1186/s13104-018-3631-z (PMC6064166; doi:10.1186/s13104-018-3631-z)
Supplement: Supplementary file 5 — Additional file 5. Post hoc analyses. Post hoc analyses showing the interaction between the various predictors (chronic illness, alcohol consumption and burnout syndrome) and the outcome—recreational drug use among 852 medical and nursing students in Cameroon from January–April 2018. [file 13104_2018_3631_MOESM5_ESM.docx]

| **Variables** | **Adjusted Odds Ratio** | **95% Confidence intervals** | **p value** |
| --- | --- | --- | --- |
| **Personal relationship (Yes/No)** | 2.08 | 0.63, 6.91 | 0.231 |
| **Presence of chronic illness (Yes/No)** | 5.26 | 1.32, 20.97 | 0.019 |
| **Alcohol consumption (Yes/No)** | 5.08 | 1.54, 16.73 | 0.008 |
| **Total OLBI score** | 1.11 | 1.02, 1.21 | 0.021 |
